# Efficacy of botulinum toxin type A administration within the current therapeutic algorithm for chronic anal fissure: study protocol for the prospective multicentre observational study FISURBOT-GECO6

**DOI:** 10.1007/s10151-026-03370-0

**Published:** 2026-06-13

**Authors:** Nuria Ortega-Torrecilla, Jorge Arredondo, Franco Marinello, Maria Luisa Reyes-Diaz, Carlos Cerdán, Oscar Cano-Valderrama, Tamara Fernández, Lidia Cristóbal, Teresa Calderón, Beatriz Martín-Pérez

**Affiliations:** 1 Department of Surgery, Hospital Universitari Vall d’Hebrón, Barcelona, Spain; 2 Department of Surgery, Clínica Universidad de Navarra. Av, Pío XII 36, 31008 Pamplona, Navarra Spain; 3 Department of Surgery, Hospital Virgen del Rocío, Sevilla, Spain; 4 Department of Surgery, Hospital Universitario de La Princesa, Madrid, Spain; 5 Department of Surgery, Complejo Hospitalario Universitario de Vigo, Vigo, Spain; 6 Department of Surgery, Hospital Galdakao-Usansolo, Bilbao, Spain; 7 Department of Surgery, Hospital Universitario Marqués de Valdecilla, Santander, Spain; 8 Department of Surgery, Hospital General Universitario Nuestra Señora del Prado, Talavera de La Reina, Spain; 9 Department of Surgery, Complejo Hospitalario Universitario de Badajoz, Badajoz, Spain

**Keywords:** Anal fissure, Botulinum toxin, Botox

## Abstract

**Background:**

Chronic anal fissure is a common, benign anorectal condition associated with impaired quality of life. Despite the availability of several therapeutic options, considerable variability persists in its management, particularly regarding the role of botulinum toxin type A administration within the current treatment algorithms. Although botulinum toxin type A injection is a widely used approach to achieve chemical sphincterotomy, its real-world efficacy and safety across different clinical scenarios remain insufficiently characterised.

**Aim:**

Our objective is to evaluate the efficacy and safety of botulinum toxin type A injection for the treatment of chronic anal fissure after failure of topical therapy or lateral internal sphincterotomy in routine clinical practice settings by analysing the healing rate, continence outcomes and the need for subsequent surgical intervention in predefined clinical scenarios.

**Methods:**

FISURBOT-GECO6 (0143–2024-OBS) is a national, multicentre, prospective, pragmatic observational study. Consecutive patients with chronic anal fissure in whom botulinum toxin treatment is recommended according to routine clinical practice are eligible. Patients will be classified into three clinical scenarios: (1) chronic anal fissure with sphincter hypertonia and no risk factors for anal incontinence after failure of topical therapy; (2) chronic anal fissure with recognised risk factors for anal incontinence after failure of topical therapy; and (3) chronic anal fissure persisting or recurring at least 12 weeks after lateral internal sphincterotomy. Botulinum toxin type A administered at a dose of 50 or 100 IU, according to routine practice. Patients will be evaluated at 1, 3 and 12 months after treatment. The primary outcome is clinical healing. Secondary outcomes are anal continence, procedure-related morbidity, need for repeat treatment or surgery and patient satisfaction.

**Expected relevance:**

This study will provide prospective, real-world data on the efficacy and safety of botulinum toxin type A injection for chronic anal fissure management in clinically relevant scenarios. The results are expected to help clarify the role of this approach within current therapeutic algorithms and inform clinical decision-making in daily practice.

**Trial Sponsor:**

Spanish Society of Coloproctology (AECP).

**Supplementary Information:**

The online version contains supplementary material available at 10.1007/s10151-026-03370-0.

## Introduction

A wide range of treatment options, including hygienic-dietary measures (HDMs), topical agents, botulinum toxin and surgical options, such as lateral internal sphincterotomy (LIS), are currently available for patients with chronic anal fissure, a common benign anorectal condition affecting approximately 1.1 per 1000 person-years [1]. In addition, different management protocols are available, with variable levels of adherence to their recommendations [2–6], as reflected in substantial differences reported in several recently published international surveys evaluating clinical practice in the treatment of anal fissure [7–9].

In most cases, the treatment of chronic anal fissure is initiated with a combination of HDMs and topical agents aimed to relax the internal anal sphincter, as reported in a study based on 152 surveys from 71 hospitals in Spain [7]. One of the most commonly used treatments is 0.4% glyceryl trinitrate, which is administered as a compounded formulation or a commercial preparation every 12 h for 2 months. The survey results revealed a healing rate of nearly 40% and a treatment non-adherence rate of 27.5%; headache was the main adverse effect. As an alternative approach, calcium channel blockers, such as diltiazem or nifedipine, are frequently used, achieving similar healing rates with fewer side effects compared with glyceryl trinitrate.

In some protocols, botulinum toxin type A administration has emerged as the next therapeutic step prior to surgery [7] or even as a first-line option replacing topical ointments in cases where HDMs fail [10]. Botulinum toxin type A, which is synthesised by *Clostridium botulinum*, causes local and temporary chemodenervation of the treated muscle, facilitating fissure healing. Although the position of botulinum toxin type A administration within the therapeutic algorithm is not fully standardised, its efficacy is comparable to that of topical treatments, with a more favourable tolerability profile. Therefore, botulinum toxin type A may be particularly appropriate in patients who develop adverse effects or exhibit poor adherence to topical therapy. In some cases, repeat administration of botulinum toxin type A after several months is needed to reduce long-term recurrence rates. In addition, its use may be limited by pharmaceutical availability. In patients with chronic anal fissure treated with botulinum toxin type A, the rate of local complications (such as perianal haematoma, limited subcutaneous infection at the injection site, perianal dermatitis or pruritus) is below 5%, and treatment-related incontinence is usually temporary, as the effect of the toxin lasts for approximately 3 months and is therefore reversible over time [7, 11, 12].

Surgery, most commonly LIS, performed using either an open or closed technique, is usually offered as the final therapeutic option. Although LIS provides a high healing rate, it is associated with flatus and faecal incontinence in approximately 20% and 3–10% of cases, respectively, and with soiling in approximately 20% of cases [7, 11, 12]. The rate of faecal incontinence following LIS may be confounded by age-related weakening of the anal sphincter mechanism and may also be influenced by the extent of sphincter division – tailored to the length of the fissure or extended to the dentate line. In any case, alternative sphincter-preserving surgical techniques have been explored, such as fissurectomy, in which chronic scar tissue and stigmata of chronicity are excised, with or without the addition of an anal advancement flap or anoplasty to introduce well-vascularised tissue into the chronic ischaemic fissure. By contrast, more aggressive techniques, such as anal dilatation – digital or pneumatic – have been progressively abandoned due to a higher risk of uncontrolled sphincter injury (up to 65%) and incontinence (up to 12%). Data on the use of botulinum toxin type A following failure of LIS are limited. In such cases, contralateral LIS has been proposed in the presence of persistent sphincter hypertonia, whereas fissurectomy may be considered in the absence of hypertonia. Several international surveys have highlighted these differences between treatment algorithms [8–10, 13–15].

Given the wide variability in published results regarding botulinum toxin type A administration and the limited standardisation of its application, we have designed a pragmatic study within the framework of routine clinical practice in our setting, with the aim of providing prospective real-world data to better inform the use of botulinum toxin type A in the management of chronic anal fissure and to support its more consistent application within the current therapeutic algorithms.

## Materials and methods

### Study design

FISURBOT-GECO6 is a national, multicentre, prospective, pragmatic, observational study designed to evaluate the efficacy and safety of botulinum toxin type A within the current therapeutic algorithm for chronic anal fissure. The study will be conducted in routine clinical practice settings and follow the Strengthening the Reporting of Observational Studies in Epidemiology (STROBE) recommendations for observational studies.

The study is scientifically endorsed and promoted by the Spanish Association of Coloproctology (AECP).

#### Hypothesis

We hypothesise that botulinum toxin type A achieves a high healing rate in patients with chronic anal fissure refractory to topical internal anal sphincter-relaxing therapies and in those who fail LIS, with a low risk of anal incontinence and minimal morbidity.

### Study population and clinical scenarios

Consecutive adult patients diagnosed with chronic anal fissure refractory to conservative measures, those with recurrence after LIS and those in whom treatment with botulinum toxin type A is indicated are eligible for inclusion in the study. Chronic anal fissure is defined as a linear ulcer extending from the dentate line to the anal verge, presenting with signs and symptoms of chronicity, including a duration of more than 6 weeks, exposure of internal anal sphincter fibres, fibrotic and thickened edges and potential association with a sentinel pile or hypertrophic anal papilla. Diagnosis will be primarily clinical, based on medical history and physical examination, and may be complemented by evaluations such as anorectal manometry and endoanal ultrasound at the discretion of the treating physician.

Patients will be analysed and stratified into three predefined clinical scenarios according to treatment history and continence risk. Scenario 1 includes chronic anal fissures with sphincter hypertonia and no identifiable risk factors for anal incontinence following the failure of HDMs and topical sphincter-relaxing therapy. Scenario 2 includes chronic anal fissures with identifiable risk factors for anal incontinence following the failure of HDMs and topical sphincter-relaxing therapy. Scenario 3 includes chronic anal fissures persisting or recurring at least 12 weeks after LIS. Topical therapy was defined as treatment with nitrates or calcium channel blockers. The number of prior topical treatment attempts before botulinum toxin administration was recorded as a study variable. Patients were considered to be at risk of incontinence if they had risk factors such as female sex, obstetric history, prior anorectal surgery, sphincter injury or chronic diarrhoea. A detailed list of all variables collected in the study protocol is provided as Supplementary Material 1.

### Selection criteria

Inclusion criteria are as follows: age between 18 and 75 years, diagnosis of chronic anal fissure fulfilling the criteria described in the clinical scenarios above, indication for treatment with botulinum toxin type A within one of the predefined clinical scenarios, and available written informed consent to participate in the study.

Exclusion criteria are as follows: known hypersensitivity to botulinum toxin type A, pregnancy or breastfeeding, pre-existing diagnosis of anal incontinence, active oncologic disease or immunosuppression, and previous treatment with botulinum toxin type A for anal fissure.

### Intervention

The study will evaluate two doses. A total dose of 50 or 100 IU of botulinum toxin type A will be administered bilaterally into the intersphincteric groove in two injections in the same session. The choice of local, topical, regional or general anaesthesia, application of ultrasound guidance and administration setting (outpatient clinic, procedure room, endoscopy suite or operating theatre) will be left to the discretion of the treating surgeon and recorded as study variables. Botulinum toxin injections are administered by surgeons with experience in colorectal practice at each participating centre. This pragmatic approach aims to reflect real-world practice and to allow the evaluation of technique-related factors influencing outcomes.

### Follow-up and assessments

Patients will be evaluated during outpatient clinic visits at 1 and 3 months after botulinum toxin type A administration. A final follow-up assessment will be conducted at 12 months, either in person or by telephone consultation. At each follow-up visit, the following will be assessed: clinical healing of the anal fissure; development of de novo anal incontinence, classified as flatus incontinence, soiling, passive incontinence or urge incontinence; changes in pre-existing continence symptoms, when applicable; procedure-related complications and patient satisfaction with treatment, evaluated using a numerical scale from 0 (not satisfied) to 10 (very satisfied). Anal continence will be assessed using the Cleveland Clinic Incontinence Score [17] and the rapid assessment faecal incontinence score [18]. Depending on the clinical evolution, the need for repeat botulinum toxin type A administration or definitive surgical treatment will be recorded.

### Study definitions and outcomes

Healing is defined as the absence of fissure-related symptoms, primarily pain and bleeding, with pruritus recorded as a secondary symptom.

Complete epithelialisation is not required, provided the patient is asymptomatic, reflecting routine clinical practice. Healing will be assessed at 1, 3 and 12 months. Persistence is defined as ongoing symptoms despite adequate treatment compliance. Intolerance is defined as the inability to complete treatment due to adverse effects. Recurrence is defined as the reappearance of symptoms after initial healing. Therapeutic failure includes both persistence and recurrence, whereas therapeutic efficacy is defined as clinical healing.

The primary outcome is the overall healing rate at 1, 3 and 12 months following botulinum toxin type A administration in patients receiving treatment as one of the three predefined clinical scenarios. Secondary outcomes are the incidence and severity of anal incontinence at each follow-up visit in all groups, procedure-related morbidity, need for surgical treatment during follow-up, subgroup analyses according to the clinical scenario and baseline patient characteristics, and comparative efficacy and continence outcomes between the 50 and 100 IU dose regimens, which will be analysed in an exploratory and hypothesis-generating manner. Data will be entered into a pseudonymised electronic database by the investigator at each participating centre.

### Statistical analysis

Quantitative variables will be described using means with standard deviation, whereas qualitative variables will be described using absolute and relative frequencies. Healing rates will be estimated, along with corresponding 95% confidence intervals, using the Wald method. Binary logistic regression models will be used to evaluate factors associated with therapeutic efficacy. Statistical significance is defined as a *p* value of < 0.05. Statistical analyses will be performed using Stata statistical software (release 18; StataCorp, College Station, TX, USA).

### Sample size estimation

The estimated sample size is 330 patients, assuming an expected healing rate of 70% at 3 months after botulinum toxin type A administration [10, 14, 15], a 95% confidence level, a precision of 5% and an anticipated follow-up loss rate of 2% at 12 months.

### Ethical considerations

The present study will be conducted in accordance with the principles of the Declaration of Helsinki. All participants will provide written informed consent prior to enrolment. All data will be collected and stored in a pseudonymised manner to ensure confidentiality.

Version 1.2 of the study was approved in January 2025 by the Ethics Committee for Research with Medicines of Navarre (ref: EO_2024/18) and has been registered in the Spanish Registry of Clinical Studies (ref. 0143–2024-OBS/AEMPS 24–00157).

The authors declare no conflicts of interest related to the present study, which has not received external funding.

## Discussion

In everyday practice, chronic anal fissure remains a challenging condition, not because of a lack of therapeutic options, but due to uncertainty regarding the optimal selection and sequencing of treatments for individual patients. Although several international guidelines propose stepwise management algorithms, substantial heterogeneity persists in clinical practice, particularly regarding second-line therapies and the role of botulinum toxin type A administration [2–6].

Botulinum toxin type A administration has been widely adopted to induce reversible chemical sphincterotomy, offering the theoretical advantage of reducing internal anal sphincter tone while minimising the risk of permanent impairment of continence. However, the existing evidence highlights marked variability in healing rates, ranging from moderate to high, as well as significant heterogeneity in patient selection, toxin dosage, injection technique, outcome definitions and follow-up duration, which complicates the interpretation of results and limits the applicability of the available evidence in routine clinical practice.

Randomised trials and meta-analyses reveal that botulinum toxin type A administration achieves healing rates comparable to those of topical sphincter-relaxing agents, with a more favourable profile of side effects, while remaining inferior to LIS in terms of definitive cure. Conversely, surgical treatment is consistently associated with a higher risk of altered post-operative continence, which may be particularly relevant in patients with baseline risk factors, such as female sex, obstetric history, history of anorectal surgery and sphincter injury. Consequently, botulinum toxin type A administration is frequently selected as an intermediate therapeutic option or an alternative to surgery in patients at higher risk of incontinence.

Despite its widespread use, the precise hierarchical position of botulinum toxin type A administration within the current therapeutic algorithms has not been clearly defined. Surveys conducted in various countries have highlighted substantial variability in its indication, timing and mode of administration, even among colorectal specialists. Differences exist not only in dose and injection technique but also in clinical scenarios where botulinum toxin type A administration is preferred over repeat topical therapy or surgical intervention [7–9]. An international survey including more than 500 responses revealed significant differences in the treatment approach depending on whether the patient was managed by a general or a colorectal surgeon. Variability was also observed in the number and type of complementary investigations and use of the treatments, such as botulinum toxin type A, according to the level of sub-specialisation [8]. Similar findings were reported in a survey conducted in Oceania in 2019, although the response rate was low (44%) [9]. A consensus rate above 70% was reached in only 7 of the 19 topics analysed. The choice of first-line treatment option in young patients and healing and incontinence rates associated with botulinum toxin type A administration and LIS were among the areas of greatest disagreement [9].

The reported high variability in the management of chronic anal fissure, a highly prevalent condition, has generated considerable interest, leading the Young Group of the Spanish Association of Coloproctology to conduct a new survey to assess the current management of anal fissure in Spain [19]. The survey was completed by 68 surgeons with specialisation in colorectal disease. The survey revealed strong consensus on initiating treatment with HDMs and topical agents for 6–8 weeks. Most surgeons modified second-line treatment according to patient sex and perceived risk of incontinence. The analysis of botulinum toxin type A use revealed that it was administered after the failure of one topical treatment in 32% of cases and after the failure of two topical treatments in 63% of cases. Botulinum toxin type A was used particularly in men with a history of surgery, in women, and in both sexes following failure of LIS. The survey demonstrated marked variability in the indication and administration protocols for botulinum toxin type A. In most cases, botulinum toxin type A was administered without anaesthesia or with topical anaesthesia, either in an outpatient clinic or in the operating theatre, without ultrasound guidance; the toxin was bilaterally injected into the intersphincteric groove. A high proportion of the surveyed surgeons considered it relevant to conduct a study specifically evaluating the efficacy of botulinum toxin type A administration within the current therapeutic algorithm for chronic anal fissure [19].

These findings are consistent with the published literature illustrating the wide range in healing rates associated with botulinum toxin type A administration, ranging from 50% to 80%, depending on the definitions used for healing and the duration of follow-up [13–15]. As reflected in the 2023 survey in Spain, there is considerable heterogeneity in the methods used to administer the toxin, including unilateral versus multilateral injection, injection at the base of the fissure or at the lateral edges and injection into the internal or external sphincter or both. The range of administered doses also varies widely across studies, further complicating the standardisation of the procedure.

Some studies have compared isosorbide dinitrate ointment with botulinum toxin type A administration, revealing that botulinum toxin type A was associated with several advantages in terms of healing and adverse effects, albeit at the expense of a higher risk of incontinence. Both therapies showed similarly high recurrence rates at 1 year [10].

In one study, botulinum toxin type A administration was associated with a higher recurrence rate (relative risk, 5.83; 95% confidence interval (CI), 2.96–11.49), lower risk of incontinence (relative risk, 0.08; 95% CI, 0.01–0.59) and greater ease of administration, compared with LIS [16].

FISURBOT-GECO6 has been designed to address this gap in knowledge by evaluating the efficacy and safety of botulinum toxin in a pragmatic, multicentre, observational setting. By embedding the study within routine clinical practice and allowing variability in the administration technique and periprocedural management, this protocol aims to capture outcomes representing everyday colorectal practice. Importantly, the study will stratify patients into three clinically meaningful scenarios that reflect common decision-making pathways, including patients with low risk of incontinence, those with recognised continence risk factors and those with persistent or recurrent fissure following LIS. Several measures were implemented to minimise bias, including prospective data collection, standardised outcome definitions and adjustment for potential confounding factors in the statistical analysis. Missing data were minimised through prospective follow-up, allowing the 12-month assessment to be conducted by telephone when in-person evaluation was not feasible.

Another strength of FISURBOT-GECO6 lies in its comprehensive assessment of continence outcomes using scoring systems in combination with evaluation of patient-reported satisfaction and assessment of the need for subsequent surgical intervention. These outcomes are particularly relevant because treatment success in patients with chronic anal fissure should be defined not only by fissure healing but also by the preservation of continence and quality of life.

We acknowledge the limitations of this study protocol. The study does not include a cost or cost-effectiveness analysis, which could be of interest for future studies evaluating the broader impact of botulinum toxin in the management of chronic anal fissure. As an observational study, the protocol does not allow direct comparison with alternative treatments and residual confounding cannot be excluded. Albeit reflecting real-world practice, variability in toxin dose, injection technique and anaesthetic use may introduce heterogeneity to outcomes. However, this variability is also a key strength of the study by allowing the exploration of factors associated with treatment efficacy and safety in routine clinical practice.

In summary, the prospective multicentre FISURBOT-GECO6 study aims to provide clinically relevant evidence on the role of botulinum toxin type A administration in the contemporary management of chronic anal fissure. The results are expected to contribute to improving our understanding of its efficacy and safety across different patient profiles and inform future refinements of therapeutic algorithms in colorectal practice.

## Supplementary Information

Below is the link to the electronic supplementary material.

Supplementary file1 (DOCX 27 KB)

Supplementary file2 (DOCX 27 KB)

## Data Availability

The trial protocol is available from the corresponding author upon reasonable request. The results of the present study will be disseminated through scientific publication and presentation at specialty conferences.
