# Supplementary material for: Efficacy of botulinum toxin type A administration within the current therapeutic algorithm for chronic anal fissure: study protocol for the prospective multicentre observational study FISURBOT-GECO6
Source: Tech Coloproctol. 2026 Jun 13;30(1):134. doi: 10.1007/s10151-026-03370-0 (PMC13490238; doi:10.1007/s10151-026-03370-0)
Supplement: Supplementary file 1 [file 10151_2026_3370_MOESM1_ESM.docx]

# Supplementary Material 1. Variables collected in the FISURBOT study protocol.

## Variables analysed

Quantitative variables will be recorded as numerical data, and qualitative variables as binary (yes/no) or multiple-choice variables.

## Demographic data

- Age
- Sex
- Diabetes mellitus
- Immunodeficiency
- Number of pregnancies
- Number of vaginal deliveries
- Episiotomy
- Previous perianal surgery
- Flatus incontinence
- Liquid stool incontinence
- Solid stool incontinence
- Soiling
- Passive incontinence
- Urge incontinence
- Constipation
- Diarrhoea
- Inflammatory bowel disease
- Previous radiotherapy
- Previous anal trauma
- Sexually transmitted disease
- Multiple anal fissures

## Centre-related data

- Type of hospital (public, private)
- AECP-accredited unit (none, basic, advanced)
- Degree of subspecialisation (general surgeon or colorectal surgeon)

## Fissure-related data

- Duration of symptoms
- Type of symptoms (pain, visual analogue scale [VAS], bleeding, incontinence, others)
- Clinical sphincter hypertonia on physical examination (yes/no)
- Endoanal ultrasound (yes/no; sphincter defect; other findings)
- Anorectal manometry (yes/no; resting pressure of the internal anal sphincter, mmHg)
- Previous use of topical sphincter-relaxing agents (one or two treatments)
- Anismus
- Use of laxatives
- Use of suppositories

## Previous topical sphincter-relaxing treatment (first-line)

- Drug
- Dose
- Dosage regimen
- Duration of treatment
- Adverse effects
- Intolerance
- Treatment adherence

## Previous topical sphincter-relaxing treatment (second-line, if applicable)

- Drug
- Dose
- Dosage regimen
- Duration of treatment
- Adverse effects
- Intolerance
- Treatment adherence

## Botulinum toxin administration

- Time interval from last treatment to botulinum toxin injection
- Administered dose (50 or 100 IU)
- Dilution
- Ultrasound guidance
- Anaesthesia (none, local, sedation, regional, general)
- Setting (outpatient clinic, procedure room, endoscopy suite, operating theatre)

## 1-month follow-up visit

- Healing
- Anal incontinence (flatus, liquid stool, solid stool, soiling, passive, urge)
- Cleveland Clinic Incontinence Score
- RAFIS score
- Changes in pre-existing incontinence (improved / unchanged / worsened)
- Local complications (infection, haematoma, other)
- Patient satisfaction (0 = not satisfied, 10 = very satisfied)

## 3-month follow-up visit

- Healing
- Anal incontinence (flatus, liquid stool, solid stool, soiling, passive, urge)
- Cleveland Clinic Incontinence Score
- RAFIS score
- Changes in pre-existing incontinence (improved / unchanged / worsened)
- Local complications (infection, haematoma, other)
- Need for repeat botulinum toxin injection
- Need for lateral internal sphincterotomy (LIS)
- Patient satisfaction (0 = not satisfied, 10 = very satisfied)

## 12-month follow-up visit

- Healing
- Anal incontinence (flatus, liquid stool, solid stool, soiling, passive, urge)
- Cleveland Clinic Incontinence Score
- RAFIS score
- Changes in pre-existing incontinence (improved / unchanged / worsened)
- Need for repeat botulinum toxin injection
- Need for lateral internal sphincterotomy (LIS)
- Patient satisfaction (0 = not satisfied, 10 = very satisfied)
