# Supplementary material for: Efficacy of botulinum toxin type A administration within the current therapeutic algorithm for chronic anal fissure: study protocol for the prospective multicentre observational study FISURBOT-GECO6
Source: Tech Coloproctol. 2026 Jun 13;30(1):134. doi: 10.1007/s10151-026-03370-0 (PMC13490238; doi:10.1007/s10151-026-03370-0)
Supplement: Supplementary file 2 [file 10151_2026_3370_MOESM2_ESM.docx]

**Supplementary Material 2:** FISURBOT-GECO6 Collaborative Group.

**FISURBOT-GECO6 Collaborative Group:** Jorge Arredondo (**Clínica Universidad de Navarra. Pamplona**), Beatriz Martín-Pérez (**Complejo Hospitalario Universitario de Badajoz**), Nuria Ortega-Torrecilla (**Hospital Universitario Vall d´Hebron**), Emilio Peña (**Hospital General Universitario Reina Sofía**), Lidia Cristobal (**Hospital Universitario Marqués de Valdecilla**), Tamara Fernández (**Hospital Universitario Galdakao-Usansolo**), Sandra Veleda y José Luís Maestro (**Hospital Universitario Río Hortega. Valladolid**), Julia Piñán (**Hospital Universitario de León**), Luís Sánchez Guillén (**Hospital Universitario de Elche**), Gerardo Rodríguez León (**Hospital del Viladecans**), Marina Alarcón (**Hospital de Sagunto**), Sandra Santarrufina Martínez (**Hospital La Plana. Villareal**), Aitor Landaluce (**Hospital Alfredo Espinosa Urduliz**), Lucía Catot (**Hospital Dos de Mayo. Consorci Sanitari Integral. Barcelona**), Natalia Alonso Hernández (**Hospital Universitario Son Espases**), Romina Pena (**Hospital Clinic. Barcelona**), Alberto Carrillo Acosta (**Hospital Universitario Virgen de las Nieves. Granada**), Cristina López (**Hospital del Bierzo**), Pablo Ruíz Quijano (**Hospital Madrid Norte Sanchinarro**), Pedro Antonio Pacheco Martínez (**Hospital Universitario Infanta Elena**), Aránzazu Calero-Lillo (**Hospital Esperit Sant. Santa Coloma de Gramanet**), Elena Hurtado Caballero (**Hospital General Universitario Gregorio Marañón**), Leticia Martín (**Hospital Talavera de la Reina**), María Fernández Hevia (**Hospital Álvarez Buylla**), Sandra Alonso (**Hospital del Mar. Barcelona**), Yolanda Ribas (**Consorci Sanitari de Terassa. Barcelona**), Marta Paniagua (**Complejo Hospitalario de Pontevedra**), Carlos Pastor (**Clínica Universidad de Navarra. Madrid**), Eloy Maldonado (**Hospital Universitario Doctor Josep Trueta. Girona**).
